# Food preference acquired by social transmission is altered by the absence of the olfactory marker protein in mice

**DOI:** 10.3389/fnut.2022.1026373

**Published:** 2022-11-09

**Authors:** Aurélie de Vallière, Ana Catarina Lopes, Andrea Addorisio, Noah Gilliand, Monique Nenniger Tosato, Dean Wood, Julien Brechbühl, Marie-Christine Broillet

**Affiliations:** Department of Biomedical Sciences, Faculty of Biology and Medicine, University of Lausanne, Lausanne, Switzerland

**Keywords:** olfaction, food preference, odorant, OMP, PS6, c-Fos, behavioral analysis, STFP

## Abstract

Food preference is conserved from the most primitive organisms to social animals including humans. A continuous integration of olfactory cues present both in food and in the different environmental and physiological contexts favors the intake of a given source of food or its avoidance. Remarkably, in mice, food preference can also be acquired by olfactory communication in-between conspecifics, a behavior known as the social transmission of food preference (STFP). STFP occurs when a mouse sniffs the breath of a conspecific who has previously eaten a novel food emitting specific odorants and will then develop a preference for this never encountered food. The efficient discrimination of odorants is performed by olfactory sensory neurons (OSNs). It is essential and supports many of the decision-making processes. Here, we found that the olfactory marker protein (OMP), an enigmatic protein ubiquitously expressed in all mature olfactory neurons, is involved in the fine regulation of OSNs basal activity that directly impacts the odorant discrimination ability. Using a previously described *Omp* null mouse model, we noticed that although odorants and their hedonic-associated values were still perceived by these mice, compensatory behaviors such as a higher number of sniffing events were displayed both in the discrimination of complex odorant signatures and in social-related contexts. As a consequence, we found that the ability to differentiate the olfactory messages carried by individuals such as those implicated in the social transmission of food preference were significantly compromised in *Omp* null mice. Thus, our results not only give new insights into the role of OMP in the fine discrimination of odorants but also reinforce the fundamental implication of a functional olfactory system for food decision-making.

## Introduction

The ability to develop a food preference is conserved from the most primitive organisms such as *Caenorhabditis elegans* (*C. elegans*) to social animals like rodents and humans (1–4). Depending on the species strategy, this behavior could be innate or learned through life experiences (5–7). As such, mammals continuously integrate olfactory cues present both in food and in the different environmental and physiological contexts to subsequently favor a given source of food with hedonic value or to avoid it (4, 8–10). In mice, for example, unfamiliar food-related odorants are naturally avoided (4) such as the detection of spoiled food-related odorants. Remarkably, food preference can also be acquired by olfactory communication in-between conspecifics, an evolutionary feature that allows the improvement of food choice performances, the so-called “social transmission of food preference” or STFP (11, 12). Behaviorally, STFP occurs when an “observer” mouse performs oronasal investigations of the “demonstrator” conspecific who has previously eaten a novel food with novel scents. The concomitant olfactory detection of these unfamiliar food-related odorants enriched with the endogenously produced carbon disulfide gas (CS_2_) of the breath then allows the development of a food preference for this demonstrated food in the observer mouse (11, 12).

**FIGURE 1:**
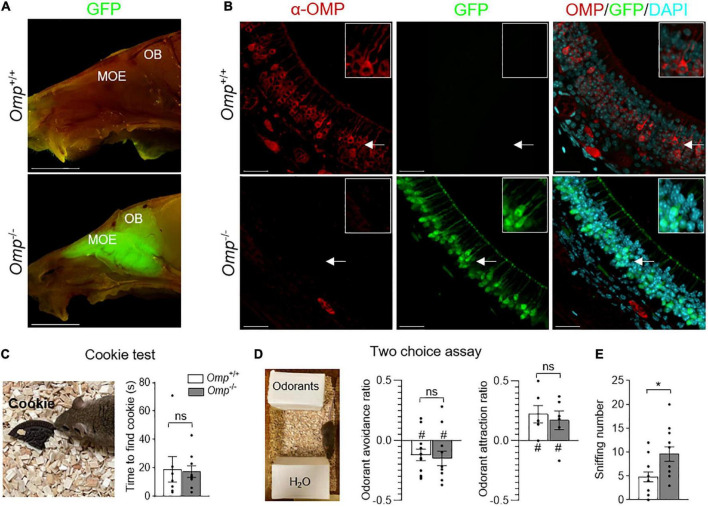
Olfactory responses observed in OMP null mice. **(A)** Sagittal cuts trough the heads of *Omp*^+/+^ and *Omp*^–/–^ mice, where GFP replaces OMP as the histological reporter of mature olfactory sensory neurons. The main olfactory epithelium (MOE) contains olfactory sensory neurons that project to the olfactory bulb (OB) of the brain. Scale bars: 1 cm. **(B)** α-OMP immunostaining of the MOE of *Omp*^+/+^ and *Omp*^–/–^ mice showing the absence of OMP expression in *Omp*^–/–^ mice. In red, the OMP expression in *Omp*^+/+^ mice. In green, the GFP expression in *Omp*^–/–^ mice. In blue, the nuclei stained with DAPI. Insets show OSNs details indicated by white arrows on main pictures. Scale bars: 20 μm. **(C,D)** The general olfactory function was not affected in *Omp*^–/–^ mice. **(C)** In a cookie test, no significant difference is observed between the *Omp*^+/+^ (in white) and *Omp*^–/–^ (in gray) mice in the latency to find an odorant Oreo^®^ cookie hidden in the bedding. **(D)** In two choice assays, tested odorants are placed opposite to the odorless water (H_2_O), both genotypes displayed similar innate odorant avoidance (butyric acid) or attraction (peanut butter solution). **(E)** Olfactory behavioral compensation observed in *Omp* null mice. *Omp*^–/–^ mice show a significant increase of sniffing behavior during the exploration of the innate odorant attraction assay **(D)**. *N* = 6–12 animals were used per genotype and condition. Values obtained are represented as mean ± SEM. For odorant avoidance/attraction *z* values, #*p* < 0.05. For comparisons between genotypes, two-tailed Student’s *t*-tests/Wilcoxon *w*-tests are used, *p < 0.05 and ns for non-significant.

The precise development of a food preference is therefore initiated by the specific recognition of odorants (4). Functionally, food-related odorants are initially recognized by olfactory sensory neurons (OSNs) located in the main olfactory epithelium (MOE) (Figure 1A). Due to their specific expression of olfactory receptors (ORs), OSNs are highly specialized sensory cells, tuned to recognize a limited set of odorants (13). Therefore, the MOE activity map is directly dependent on odorant detection thanks to the associated ORs recognition. An encoding strategy that is also found in the first olfactory brain relay center, the olfactory bulb (OB) (Figure 1A) as OSNs expressing the same OR project to the same specific OB subregions named glomeruli (13–15). Consequently, each food-related cue found in the demonstrator breath, would be parallelly processed by hardwire circuitries emerging from OSNs. The endogenous CS_2_, for example, would thus be initially detected by guanylyl cyclase-D (GC-D) expressing OSNs that project to phosphodiesterase 2A (PDE2A) positive necklace glomeruli structure (12, 16) before being integrated by different brain regions to subsequently develop food preferences (4).

Genetic approaches have shown that the deletion of specific odorant receptor genes led to particular food preference alterations (17). On the other hand, downstream signaling elements that are shared by all ORs-expressing neurons such as the adenylyl cyclase of type III (ACIII) or the cyclic nucleotide-gated (CNG) calcium channels (15) are crucial for fundamental olfactory functions as their genetic deletion generally leads to global and severe phenotypes such as anosmia (18). Pups from transgenic mice models with non-functional CNG channels, for example, are unable to find their mother nipples and thus die early (9, 19, 20).

Interestingly, the deletion of the enigmatic olfactory marker protein (OMP) which is expressed in all mature OSNs (21) and highly conserved throughout evolution (22), argues for a fundamental olfactory role, only leads to limited phenotypes. Indeed, in these *Omp* null mice models, different impairments were observed such as, limited signal transduction defects at the olfactory sensory neuron level (23, 24), a general decrease in odorant sensitivity and discrimination (25), a restricted glomerular mistargeting, a decrease in the specific glomerular response (26) and the absence of mother-related milk preference (21, 23, 27–30).

While mapping the OSNs-related odorant activity in an *Omp* null mouse model (31), we found that the absence of OMP induced a higher basal neuronal activity both in the MOE and in the OB. Although these mice were still detecting odorants and their hedonic values, we observed that they displayed a considerably reduced olfactory performance during behavioral assays involving fine discrimination of complex odorant signatures such as the ones found in social and food-related contexts. We finally demonstrated that OMP is an essential molecular determinant implicated in the social discrimination and acquisition of a food preference. Thus, our results not only give new insights into the role of OMP in olfaction but also reinforce the fundamental implication of the olfactory system in the development of food decision-making.

## Results

### Olfactory marker protein regulates the basal olfactory activity of main olfactory epithelium neurons

Using an OMP-GFP mouse model (31; Figure 1A), we observed, in the mouse head, a GFP signal exclusively localized in the olfactory system. It was correlated with the absence of OMP expression (Figure 1B) as immunohistochemistry approaches revealed the expression of OMP in *Omp*^+/+^ (GFP negative) in MOE neurons, while its total absence was observed in *Omp*^–/–^ (GFP positive) mice. We next verified the ability of these null mice to smell general odorants with a hidden food test [the buried cookie test (32, 33)] and found that *Omp*^+/+^ and *Omp*^–/–^ littermates were equally efficient (Figure 1C), as previously observed (21, 29). Moreover, challenging mice in a two choice assay where hedonic-associated odorants were opposed to the odorless water (4), we observed that *Omp* null mice still displayed innate avoidance (food spoiled with butyric acid) and attraction (peanut butter) (Figure 1D) for given odorants. Nevertheless, we noticed that the overall sniffing behavior performed by these *Omp* null mice was significantly increased (Figure 1E), arguing for an olfactory compensatory adaptation.

**FIGURE 2:**
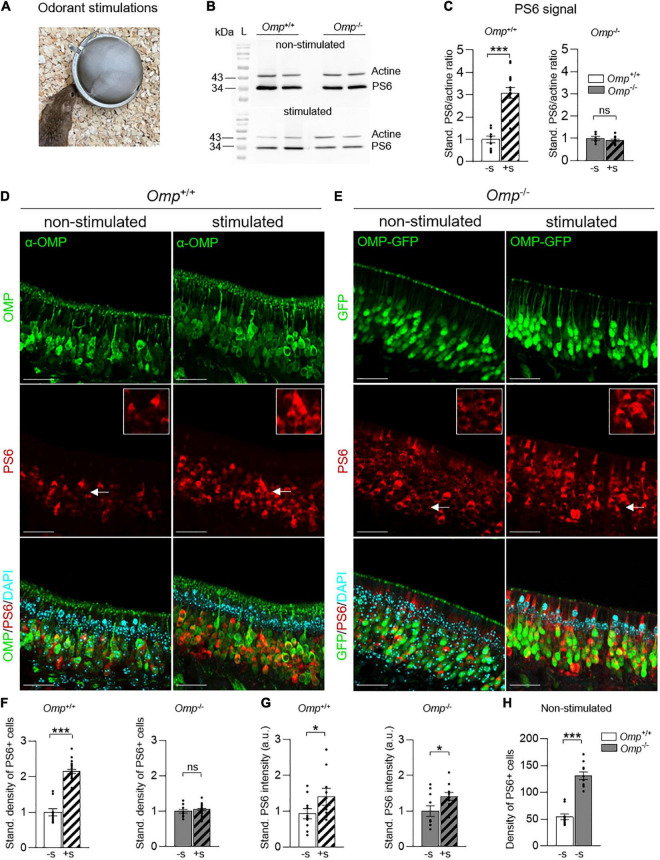
Increased basal activity of MOE neurons in OMP null mice. **(A)** Odorant stimulations are performed by using odorant solutions (Cinnamon 10% or Cocoa 20%) or the odorless water, during 1 h. **(B)** Western blot of phosphoserine 6 (PS6). At 32 kDa, the bands represent the PS6 expression and at 43 kDa, the actine expression (control). The results show the intensity difference of PS6/actine in *Omp*^+/+^ and *Omp*^–/–^ mice in stimulated (here, with Cinnamon) and non-stimulated conditions (water). L, ladder. **(C)** Western blot analysis of the PS6 signal in the MOE of *Omp*^+/+^ and *Omp*^–/–^ mice with (+S; dashed bars) or without (–S; solid bars) odorant stimulation (obtained from Cinnamon and Cocoa extracts) showing the PS6/actine standardized ratio. *N* = 5–9 animals were used per genotype and condition **(B,C)**. **(D,E)** PS6 immunostainings in the MOE of *Omp*^+/+^ **(D)** and *Omp*^–/–^ **(E)** with or without odorant stimulation. In red, the staining of PS6 shows the activated neurons. In green, the staining of mature olfactory sensory neurons. In blue, the nuclei stained with DAPI. Insets show OSNs details indicated by white arrows on main pictures. Scale bars: 30 μm. **(F)** Odorant stimulation (+S; here, with Cinnamon) induces a significant increase of activated neurons in *Omp*^+/+^ but not in *Omp*^–/–^ mice. **(G)** PS6 staining intensity is increased significantly in the stimulated condition compared to the non-stimulated condition for both the *Omp*^+/+^ and *Omp*^–/–^ mice. Dot-plots representation in panels **(F,G)** are standardized to the non-stimulated condition −S. **(H)** Spontaneous basal neuronal activity is increased in *Omp*^–/–^ mice as shown by the density of the observed PS6 positive cells [insets in panels **(D,E)**]. *N* = 5 animals were used per genotype and condition. Values obtained are represented as mean ± SEM. For comparisons between conditions and between genotypes, one-tailed or two-tailed Student’s *t*-tests/Wilcoxon *w*-tests are respectively used, *p < 0.05, ***p < 0.001 and ns for non-significant.

We next looked at the general OMP expression in their olfactory system, focusing first on the MOE and challenging the observation of OSNs activity from a rostral detection to a brain integration at the level of the OB. For that, we took advantage of the serine 240/244 phosphorylation phenomenon that takes place on the ribosomal protein S6 when OSNs are stimulated by odorants (34). Accordingly, we then used phosphoserine 6 (PS6) as a neuronal marker of OSNs activities (34–36). We exposed mice (Figure 2A) to the odorless water (Non-stimulated; Figure 2B) or to the complex odorant mixtures of Cinnamon (Stimulated; Figure 2B) and Cocoa extracts (Stimulated; Supplementary Figure 1). We found, by Western blot analysis, a significant increase of the PS6 signal in *Omp*^+/+^ corresponding to an intensified OSNs activity after odorant stimulations *vs.* the non-stimulated condition (Figures 2B,C and Supplementary Figure 1). Surprisingly, no significant increase in activity was observed in *Omp*^–/–^ littermates (Figures 2B,C and Supplementary Figure 1). To further study this observation, we focused on the PS6 responses observed at the MOE tissular level performing immunostaining investigations on both genotypes (Figures 2D,E). With this approach, we confirmed our previously observed increase of PS6 signal, as the density of PS6-positive OSNs were significantly upregulated in *Omp*^+/+^ mice after odorant stimulation (Figure 2F) and we also saw its alteration in *Omp*^–/–^ compared to the non-stimulated condition (Figure 2F). Nevertheless, in both genotypes, the PS6 signal intensity was significantly upregulated after odorant stimulations (PS6 inset; Figure 2G), indicating that a neuronal stimulation was still achieved in *Omp*^–/–^ mice (Figure 2G) and confirming the ability of the null mice to detect general odorants (26) as observed on the buried cookie test (Figure 1C). Conversely, and compared to the *Omp*^+/+^ littermates, we noticed a significant increase of PS6-positive OSNs in *Omp*^–/–^ mice under non-stimulated condition (Figure 2H). This indicated a striking and higher general basal activity level of the MOE neurons in the absence of OMP. Taken together, our results strongly suggest that, in the absence of OMP, mice still display olfactory sensing abilities with an altered PS6 response linked to a higher basal activity of the MOE. Hence, we found that OMP regulates the basal activity of mouse MOE neurons.

### Olfactory marker protein regulates the odorant integration in the olfactory bulb

**FIGURE 3:**
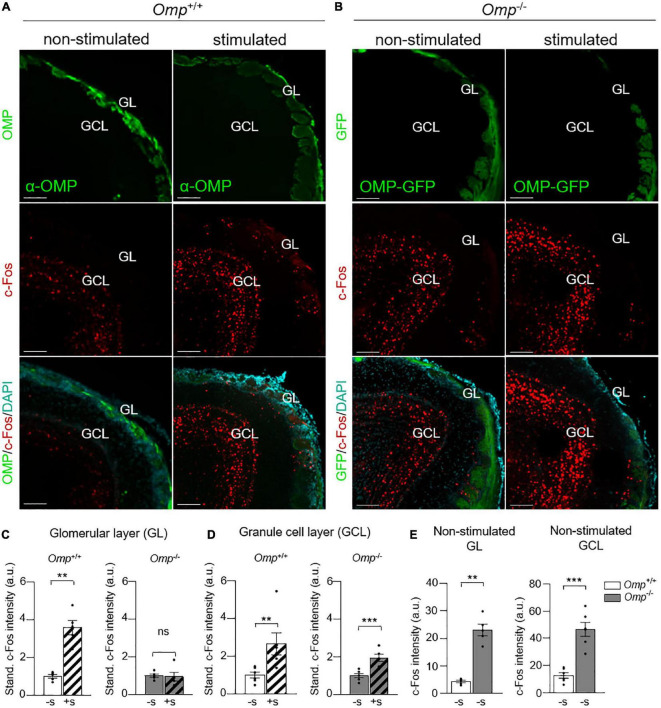
Functional integration of odorants in OMP null mice. **(A)** c-Fos immunostainings in the olfactory bulb of *Omp*^+/+^ **(A)** and *Omp***^–^**^/^**^–^** mice **(B)** with or without odorant stimulation (here, Cinnamon). In green, the staining of mature olfactory sensory neurons. In blue, the nuclei stained with DAPI. GL, glomerular layer; GLC, granule cell layer. Scale bars: 100 μm. **(C)** In the glomerular layer, a significant increase of the c-Fos staining intensity after odorant stimulation (+S; dashed bars) is only observed in *Omp*^+/+^. **(D)** In the granule cell layer, a significant increase of the c-Fos staining intensity is observed in both genotypes after odorant stimulation. Dot-plots representation in panels **(C,D)** are standardized to the non-stimulated condition (–S; solid bars). **(E)** An increased basal bulbar activity is observed in *Omp*^–/–^ mice as shown by the intensity of c-Fos positive cells both in the GL and GCL. *N* = 7–9 animals were used per genotype and condition. Values obtained are represented as mean ± SEM. For comparisons between conditions and between genotypes, one-tailed or two-tailed Student’s *t*-tests/Wilcoxon *w*-tests are respectively used, **p < 0.01, ***p < 0.001 and ns for non-significant.

To further assess the integrative part of the observed PS6-responses in the MOE, we next used c-Fos staining as a neuronal marker of OB activity (37). After odorant stimulations, we generated OB slices on which we performed immunohistochemistry investigations. We then focused on the different c-Fos signal intensity observed both in the glomerular layer with the first OSNs relay, the OB glomeruli (GL) (Figures 3A,B), as well as in the OB integrative granule cell layer (GCL) (Figures 3A,B; 26). As expected, we observed an increase of c-Fos signal intensity under odorant stimulation in *Omp*^+/+^ mice (Figures 3A,B) not only in the glomerular layer (Figure 3C) but also in the granule cell layer (Figure 3D). On the other hand, *Omp*^–/–^ mice showed a significant increase in c-Fos signal only in the granule cell layer (Figure 3D), indicating that even in absence of OMP, mice still displayed odorant integration. We next compared the c-Fos responses between both genotypes under non-stimulated condition. Interestingly, we noticed, in *Omp*^–/–^ mice, approximately 10% of additional basal active glomeruli as well as a significant higher intensity of c-Fos signal both in the glomerular and granule cell layer (Figure 3E) not only reinforcing previous observations (26) but also confirming our MOE data (Figure 2E). Our results show that OMP regulates the threshold of detection and integration of olfactory signals possibly altering odor perception (21, 23, 27–30).

### Olfactory marker protein is an essential molecular determinant implicated in the social discrimination and acquisition of a food-related odorant preference

**FIGURE 4:**
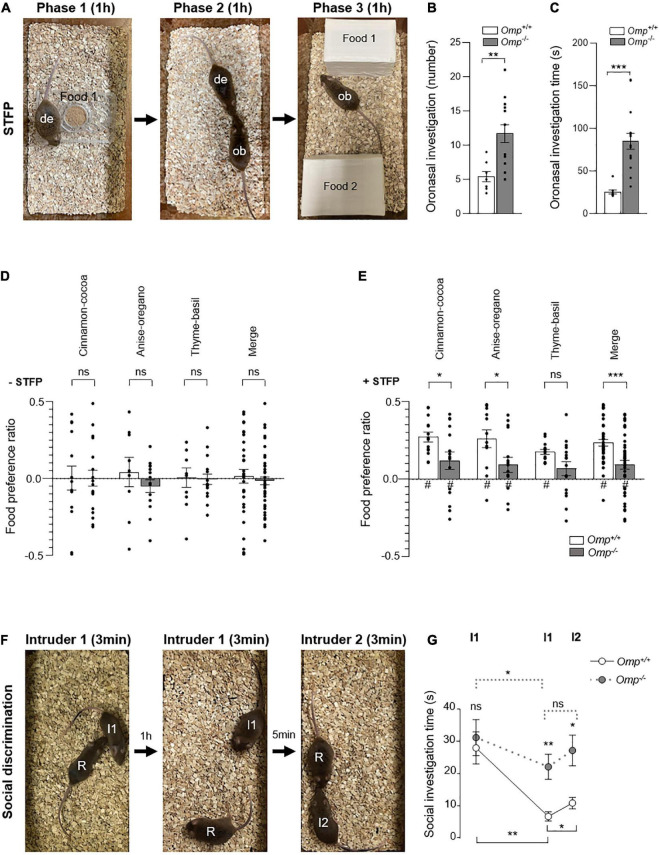
Altered social transmission of food preference and discrimination in OMP null mice. **(A–E)** Social transmission of food preference (STFP) assay. **(A)** The demonstrator mouse (de) eats the demonstrating food (Food 1; Phase 1) during 1 h before it returns with its littermates. Then, oronasal interactions between the demonstrator (de) mouse and an observer (ob) mouse to transmit olfactory information about the novel food (Phase 2) are performed during 1 h. Then, the ob mouse is faced with the two choice assay (Food 1 *vs.* Food 2; Phase 3) during 1 h. The *Omp*^–/–^ mice (in gray) show a significant increase in the number **(B)** and the duration **(C)** of oronasal investigations compared to *Omp*^+/+^. **(D)** In absence of STFP procedure (–STFP), both mice genotypes show no innate preference for the three couples of spices used: Cinnamon-Cocoa, Anise-Oregano and Thyme-Basil when presented in a counterbalanced mode. **(E)** After STFP (+ STFP), *Omp*^–/–^ mice showed a significantly altered acquisition of food preferences for each of the three tested couples of spices as well as in the merged data (Merge). **(F,G)** Social discrimination test. **(F)** The social investigation performed by a Resident (R) mouse is observed when a never encountered before Intruder 1 (I1) is introduced into the cage during 3 min. After 1 h, Intruder 1 is reintroduced into the cage and the social investigation time performed by the Resident mouse is again quantified during 3 min. After 5 min, another Intruder mouse (I2) is then presented and the 3 min of social exploration time performed by the Resident mouse are quantified. **(G)** Statistical analysis of the social investigation time performed in panel **(F)** for *Omp*^+/+^ (white circles and continuous line) and *Omp*^–/–^ mice (gray circles and dashed line) mice. *N* = 6–16 **(B–E)** and 6–7 **(G)** animals were used per condition and genotype. Values obtained are represented as mean ± SEM. For food preference *z* values, #*p* < 0.05. For comparisons between genotypes, two-tailed Student’s *t*-tests/Wilcoxon *w*-tests were used; for comparisons between conditions **(G)**, one-tailed paired Student’s *t*-tests were used, *p < 0.05, **p < 0.01, ***p < 0.001 and ns for non-significant.

To further assess the ability of *Omp*^–/–^ mice to finely distinguish odorant complexity, we next took advantage of the STFP assay as this behavior requires relevant olfactory discrimination both in food and in a social context (4). To do this, the demonstrator mice first ate individually a never encountered demonstrated food (a standard powdered food odorized with spice #1, for example Cinnamon, as Food 1; Figure 4A) for 1 h (4). We first found that both genotypes were equally efficient in performing this initial passive conditioning period, as no difference were found in their food consumption (*Omp*^+/+^, N = 21, 0.63 ± 0.06 g; *Omp*^–/–^, N = 21, 0.52 ± 0.03 g; ns). Then the demonstrator mice were returned to their littermates to allow the STFP process to happen (Phase 2; Figure 4A). During this phase, we noticed that the oronasal interactions performed by the observer mice to detect these unfamiliar and Food 1-related odorants in the demonstrator breath were affected by the absence of OMP, independently of the demonstrator mouse genotype (Figures 4B,C). Indeed, both the number of contacts (sniffing/touching the mouth/face area) (Figure 4B) and the total time spent (Figure 4C) to perform these oronasal investigations (38) were significantly increased in *Omp*^–/–^ mice, supporting our initial olfactory compensation observations (Figure 1E). Moreover, to exclude the potential absence of breath/CS_2_ detection in *Omp*^–/–^ mice, we confirmed by immunohistochemistry that the null mice also possess the GC-D hardwired circuitry (39) both in the MOE and OB (PDE2A-positive OSNs and glomeruli; Supplementary Figures 2A,B) as well as its functionality (CS_2_-dependent c-Fos activity in the OB necklace glomeruli; Supplementary Figures 2C–G). These results further supported our hypothesis on the role of OMP in odorant discrimination. We next challenged the mice in a two choice assay (Phase 3; Figure 4A) giving them free access to two odorized foods, the demonstrated food (Food 1; odorized with spice #1, for example Cinnamon) and a novel food (Food 2; odorized with spice #2, for example with Cocoa), presented in counterbalanced mode to avoid any individual innate preference (–STFP; Figure 4D; 4, 40). Interestingly, after STFP, we found that both genotypes were able to display food preferences with different sets of odorized food (Cinnamon—Cocoa, Anise—Oregano or Thyme—Basil, used as spices #1 or #2 in a counterbalanced mode; +STFP; Figure 4E). Nevertheless, we noticed a significant alteration in the behavior performed by *Omp*^–/–^ mice, demonstrating that despite an intensified oronasal investigation observed in these mice (Figures 4B,C), their efficiency to display food-related odorant choices remained altered compared to their littermates *Omp*^+/+^ (Figure 4E).

To determine whether *Omp* null mice have a specific deficit in STFP/food-related odorant recognition rather than an alteration in their general olfactory capacities, we next examined the social discrimination ability displayed by these mice in a resident—intruder paradigm assay (41; Figures 4F,G). For that, a resident mouse (R) (Figure 4F) socially investigates a first intruder mouse (I1) (Figure 4F). The total time of its anogenital, nose-to-nose sniffing and allogrooming is quantified during the first 3 min session (Figure 4G). As expected, no significant difference was observed, during this period, between both genotypes, confirming the sociability of *Omp* null mice. To further characterize the social memory performance, the intruder 1 is presented again after 1 h to the resident mouse during another 3 min session (Figure 4F). We observed that both genotypes were able to show habituation toward intruder 1, as the total investigation time significantly decreased across sessions (Figure 4G), which correlates with the absence of OMP expression in the memory-associated brain regions (Figure 1A). Nevertheless, compared to their wildtype littermates, *Omp* null mice investigate significantly more the intruder 1 in the second session (Figure 4G). To further confirm, that this observed investigation impairment was related to an alteration in the olfactory-related discrimination task rather than in the recall memory process, we next presented a second intruder 2 (I2) (Figure 4F) to the resident mouse. Contrary to their *Omp*^+/+^ littermates, *Omp* null mice indeed failed to efficiently discriminate between both intruders as they did not show a significant increase in their social investigation time (Figure 4G and Supplementary Figure 3).

Based on these mice behavioral assays, we thus confirmed that OMP is essential for the discrimination of complex odorant signatures. We further demonstrated the relevance of OMP to perform an efficient social discrimination and food-related odorants preference previously acquired by social transmission.

## Discussion

In the wild, the ability to precisely decipher the environment is essential and allows social animals to fulfill their physiological and safety needs that not only ensure the individual survival but also increases the overall fitness of the species (4, 42). In stressful environmental conditions, such as predation or starvation, conspecifics cohesion and interactions are thus especially required (43, 44). To ensure these fundamental tasks, rodents have developed elaborated behavioral strategies and used a particularly sophisticated sense of smell (4). In mice, the fine discrimination of social-related odorants thus not only ensure the recognition of its own belonging but also allows to share knowledge in-between conspecifics. The ability to socially transmit information about food quality is, as such, a key advantage not only to prevent poisoning and sickness (45, 46) but also to evaluate efficiently the benefit to eat a novel source of food (4, 12, 47–49). Overall, our results demonstrate that OMP regulates the basal neuronal activity of MOE and OB neurons. Indeed, its absence alters the ability of the mice to perceive complex odorant signatures found for example in conspecifics or in the food and thus prevents the efficient development of a food preference. Accordingly, we can therefore speculate that the high genetic conservation of OMP observed throughout evolution (22) could be partially explained by the olfactory discrimination advantage conferred by the expression of this protein. Interestingly, in humans, a 88% conserved OMP (22), which is also an indicator of olfactory neuronal maturity, is already present by the 28–29th week of gestation (50, 51). It has been shown that human mothers influence the hedonic polarity of their neonates’ initial olfactory responses and acquisition of food preference through their diet (52). OMP could thus participate in these early influences of general social cognition and therefore in the development of food preferences in humans (53).

In this study, we investigated the PS6-related signal to assess the global MOE neuronal activity (34). Interestingly, the absence of OMP induced a higher basal activity in this olfactory subsystem. This phenomenon could be further investigated by physiological techniques such as imaging approaches (26, 39), as its precise functional process remains elusive. Nevertheless, using a parallel approach, we confirmed the biological relevance of our PS6-related results by c-Fos investigations at the OB level. Remarkably, odorant detection was still observed both at the tissular (MOE neurons) and behavioral levels even in the presence of this phenomenon, thus raising the question of how a higher basal activity could yet impact the olfactory discrimination process in these null mice. An elegant explanation lies in the observation that, in *Omp* null mice, the number of active OSNs was not increased following odorant stimulations but that intensity of the response itself was thus reinforcing previous electrophysiological observations made at the individual OSNs level where depolarization signals were amplified (20). This could therefore suggest that the olfactory system of *Omp* null mice would be continuously active in the absence of odorants. As a consequence, the MOE and the OB neuronal activation maps would be modified by this reminiscent odorant background which would impact olfactory encoding (13). Reinforcing this assumption, we noticed 10% additional basally active glomeruli in the OB of *Omp* null mice. An encoding alteration that results in an absence of significant increase of c-Fos intensity after odorant stimulations in this layer. Interestingly and in spite of the dilution effect of this glomeruli-related activity, the odorant information appeared to be integrated as a significant increase of activity was observed in the granule cell layer. This phenomenon, coupled with the other functional defects attributed to the absence of OMP, such as in the signal transduction (27, 29), in the ORs expression (27) or in the cAMP regulation (54, 55), would therefore be all the more significant when the animals are exposed to complex mixtures of odorants such as the ones present in conspecifics (social investigations/odor discrimination test) or in food as displayed in the STFP process (oronasal investigations/two choice assays). As a consequence, we observed that *Omp* null mice have to compensate by increasing their sniffing behavior not only for sensing their surroundings (innate avoidance and attractive tests), but also during STFP (oronasal investigations) and social investigations probably linked to the necessity to reach and to get more odorant-related information to finalize their decision-making.

In the course of our investigation, we focused our attention on the MOE. Nevertheless, the olfactory system of the mouse is complex and composed of specialized olfactory subsystems that all express the OMP such as the vomeronasal organ (VNO) and the Grueneberg ganglion (GG) respectively implicated in pheromonal and danger-related odorant detection (39, 56). Further investigations of the specific or ubiquitous role(s) of OMP in the VNO or in the GG could be performed to verify whether the phenomenon observed at the level of the MOE takes place in all the OSNs composing the olfactory system. Hence OMP could also regulate pheromonal and kairomonal detection and thus communications in-between conspecifics (*via* VNO: non-volatile cues; or *via* MOE: volatile cues) and heterospecifics (*via* GG: volatile cues) the same way it affects food odorant recognition (*via* MOE: volatile cues) (9, 19, 20). Accordingly, researchers using this very useful OMP-GFP model rationally undertake standard control experiments, such as experimental validations on control wildtype mice (21, 31). A precaution that should continue in absence of this sensory information.

Olfactory marker protein is exclusively expressed in OSNs and not in higher brain regions implicated in long term memory (4, 31). While, we observed that *Omp* null mice still developed a social memory, their performances were nonetheless altered compared to wildtype littermates. We interpreted these results as a consequence of an impairment in social-related odorants discrimination rather than of a recall memory defect. However, we cannot totally rule out the OMP’s involvement on memory processing in particular for STFP process. Further experiments based on *Omp* null mice and shorter/longer memory STFP paradigm (40), could therefore contribute to resolve this elusive issue.

The complex hardwired circuitries initiated by an odorant activating the OSNs signal transduction is then transmitted into the OB for further deeper cerebral incorporation. Parallel integrations emerging from different sensory inputs take place in the brain to generate a final behavior that allows mice to deal with their environment and physiological needs. Here, using a mouse model in which the OMP expressed exclusively in the olfactory system was genetically deleted, we highlighted the importance of this molecular determinant in the rostral detection and discrimination of both social and environmental cues, as well as in the integration of the odorant signal and the development of a food preference.

## Materials and methods

### Animals

Male and female OMP-GFP (31) littermates, obtained by crossing heterozygous mice, were used. In this gene-targeted knock-in mouse strain OMP-GFP, GFP replaces OMP as the histological reporter of mature olfactory sensory neurons (30) expressed under the control of the OMP promoter (28, 31, 57). Mice were housed in the animal facility and then in an independent behavioral room under mentioned (standard or reverse) light-dark cycle. Sacrifices were performed under CO_2_ or cervical dislocation. The experimental procedures were in accordance with the Swiss legislation and approved by the EXPANIM committee of the Lemanique Animal Facility Network and the veterinary authority of the Canton de Vaud (SCAV).

### Genotyping

Mouse DNA was extracted and amplified according to alkaline extraction and polymerase chain reaction (PCR) procedures (58). Briefly, PCR was conducted with the following primers: *Omp*^+/+^ (forward 5′–GAAGCAGCAGCTGGAGATG–3′; reverse 5′–GCATCCGGC TTCTAGACT–3′); *Omp*^–/–^ (forward 5′–CAGCGTGCAGCT CGCCGACC–3′; reverse 5′–GCAGCATCCGGC TTCTAGA CT–3′). PCR products and 100bp-DNA ladder (Bench Top G829B, PROMEGA, Dübendorf, Switzerland) were then loaded on an 2% agarose gel supplemented with ethidium bromide. Standard electrophoresis followed by DNA revelation under UV light were performed.

### Preparation of odorant solutions and stimulations

Infusion of Cinnamon 10% (McCormick, MA, USA) and Cocoa 20% in distilled water were prepared by warming the solution at 45–50°C under a constant agitation for 1 h. Infusions were then centrifuged at 12,000 rpm during 5 min and supernatants were collected and stored at 4°C (<1 day) for further use. Odorant stimulations were performed by using odorant solutions (Cinnamon 10% or Cocoa 20%) delivered on a cotton swab and placed with mice during 1 h. Similarly, CS_2_ stimulation (10 ppm, Merck, Aubonne, Switzerland; #335266) was performed for the specific stimulation of GC-D circuitry (4). For each condition, 2 × 500 μl of odorant solutions were used. Mice were then sacrificed for further analysis.

### Western blot analysis

Western blotting was performed in order to detect the PS6 protein after odorant stimulations. Mice were sacrificed, the MOE was extracted and immediately frozen in liquid nitrogen and stored at –80°C until use. Then, the whole MOE extracts were prepared (59) using RIPA lysis buffer (Tris 50 mM pH 7.2, NaCl 150 mM, NP40 1%, SDS 0.1%, Na-deoxycholate 0.5%), protease inhibitor mixture (Pepstatine A, Aprotinin, Leupeptin hemisulfate, PMSF, from Sigma, Aubonne, Switzerland) and phosphatase inhibitor cocktail (Thermo Fisher Scientific, MA, USA). The concentration of the isolated proteins was determined using BCA Protein Assay Reagent (Pierce BCA Protein Assay Kit, Thermo Fisher Scientific, MA, USA). Then, 25 μg of the proteins were separated on a 13% Tris-acetate gel and electrophoretically transferred to membranes (Amersham Protran 0.2 μm NC, GE Healthcare Life science, Zürich, Switzerland) with BioRad apparatus. Membranes where then incubated with the primary antibody Rabbit anti-PS6 (1/5,000, Cell signaling, MA, USA; #5364) or Rabbit anti-β-actin (1/2,500, Merck, Aubonne, Switzerland; #A2066) and finally with the appropriate horseradish peroxidase conjugated secondary antibody (Anti-Rabbit 1/10,000, Jackson ImmunoResearch, Cambridge, UK) and revealed by chemiluminescent immunodetection (SuperSignal West Pico PLUS Chemiluminescent Substrate, Thermo Fisher Scientific, MA, USA). The acquisition was performed by FusionSolo Chemiluminescence. Using the open access imageJ software (National Institute of Health, 1.48 v), an intensity counting approach was performed for each PS6 protein extraction band obtained. An average of minimum 4 MOEs was used for the global protein extraction of PS6. The western blots were realized on 4–7 animals of each genotype and experimental condition.

### Immunohistochemistry

Immunostainings were performed both on cryosections (HM 525NX; Thermo Fisher Scientific, MA, USA) and floating sections (VT1200S; Leica) according to the tissular morphology (60). Briefly, after 4% PAF (paraformaldehyde 4%, pH 7.6) fixation phases, mouse heads were transferred for 24 h in a 0.5 M EDTA (Ethylenediaminetetraacetic acid, pH 8.0) decalcifying solution. MOE and OB were then precisely dissected and collected before being placed in OCT or 4% agar block. Coronal tissue slices of respectively, 20 μm and 100–120 μm were then generated. Immunostaining procedure was initiated by a blocking/permeabilization phase with specific serum depending on the antibody species, respectively, 5% of normal donkey serum (NDS, Jackson ImmunoResearch, Cambridge, UK) or 5% normal goat serum (NGS, Interchim, Montluçon, France) and 1% Triton X-100 (TX100, Fluka analytical, Aubonne, Switzerland) for at least 2 h at 4°C. Primary antibodies were used in specific serum solution for at least 24 h at 4°C: Rabbit anti-PS6 (1/400; Cell signaling, MA, USA; #5364), Rabbit anti-c-Fos (1/500; Cell signaling, MA, USA; #2250), a Rabbit anti-PDE2A (1/400; FabGennix, San Francisco, USA), Goat anti-OMP (1/1000; Wako, Richmond, USA). After washing phases, the following secondary antibody were used for 2 h: Donkey Cy3 anti-rabbit (1/200; Jackson ImmunoResearch, Cambridge, UK), Donkey FITC anti-goat (1/200; Jackson ImmunoResearch, Cambridge, UK) and Goat 647 anti-rabbit secondary antibody (1/200; Thermo Fisher Scientific, MA, USA). Slice mounting was performed with Vectashield media containing DAPI (H-1200; Vector Lab, Servion, Switzerland). Acquisitions were done by confocal microscopy (LEICA SP5 TANDEM/LEICA STELLARIS 8, Leica Biosystems, Muttenz, Switzerland) with 10×–40× objectives and analyzed under computer assistance (v7.1.1, Imaris).

### Behavioral assays

#### The hidden cookie test

The ability to smell general odorants was tested according to the buried cookie test (32, 33). Briefly, after a 24 h-food deprivation, mice were challenged to find an Oreo^®^ cookie buried in the home cage of the subject under 1 cm of bedding. Experiments were performed on two consecutive days where the first day is the training session and the second day, the test session. To avoid place-preferences, the location of the cookie was systematically modified across assays. Latency to find the cookie was measured.

#### Innate odorant avoidance and attraction

Behavioral tests were performed in an independent behavioral room under standard light-dark cycle. Male and female mice, were tested with an equivalent sex ratio. Innate odorant avoidance and attraction were evaluated using a two choice assay (4). Mice were placed individually in a clean cage where they could freely explore an odorized source and a non-odorized source. Butyric acid (BA) and peanut butter (PB) solutions were used for innate odorant avoidance and attraction, respectively (4, 8–10). For innate odorant avoidance, 24 h food-deprived mice were challenged during 1 h to choose between two cups of food placed in the opposite part of the cage in an accessible semi-closed box (∼500 cm^3^) to limit odorant diffusion. Five hundred microliters of BA 5% (diluted in water) or the odorless water was deposited around the food without any direct contact. The consumption of each food sources was measured and used as criteria for quantification. For innate odorant attraction, a solution of 500 μl PB 10% (diluted in water) or the odorless water was deposited on opposite filter papers. The number of sniffing directed to each source was measured during 3 min and used as criteria for quantification. The quantification of odorant avoidance/attraction ratio was then calculated according to the ratio between the tested odorant *vs.* the total (odorants + water) criteria minus 0.5 (corresponding to the non-preference threshold). The values were expressed between 0.5 and –0.5, where positive scores represented an attraction and negative scores an avoidance for the tested odorant.

#### Social transmission of food preference assay

Experiments were conducted in a semi-blind approach where the affiliated experimenter was unaware of the mouse genotype. Assays were performed in an independent behavioral room under reverse light-dark cycle. Male and female mice, from both genotypes, were tested with an equivalent sex ratio per condition. Mice were tested with different sets of spices and each couple of spices was used once per mouse. The delivery of food chow was performed with homemade-ballasted cups allowing to limit food spread (4) and commercial spices (McCormick, MA, USA or local distributers) were used as dry powders and added to the mouse powdered food chow. The tested pairs of odorants (spice #1—spice #2) were: Cinnamon (1%)—Cocoa (2%), Anise (0.8%)—Oregano (2.4%) and Thyme (1.8%)—Basil (1.4%). To perform the STFP process (4, 12), 2 g of powdered food chow was first given to each mouse during 48 h followed by 24 h of food deprivation. Then, the separated demonstrator mouse ate the demonstrating food during 1 h and a consumption ≥0.2 g was necessary for further analysis (Phase 1). The demonstrator mouse was returned into its home cage with the observer littermates for 1 h of STFP learning process (Phase 2). As the success of this process was necessary for the rest of the experiment, the first 15 min of procedure were thus video-recorded for the quantification of the social interactions (40). Briefly, the mean number of oronasal (nose-mouth) contacts between the observer mice and the demonstrator mouse as well as the mean duration time spent for each contact performed during oronasal investigations were quantified according to the total number of observer mice present in the cage and the total number of social interactions respectively recorded. After this 1 h of procedure, the observer mice were individually confronted to a two choice assay (Phase 3) where the demonstrated food (Food 1; powdered food chow odorized with spice #1) and a novel food (Food 2; powdered food chow odorized with spice #2) were presented in counterbalanced mode during 1 h (spice #1 and spice #2 used equivalently as demonstrated foods) to avoid any individual innate preference (4). The cup positions were varied randomly and each source of food was given in sufficient amount (3.0–3.5 g). A consumption ≥0.2 g per observer mice was necessary for further analysis (4). The quantification of the food preference ratio for the demonstrated Food 1 was then calculated according to the ratio between Food 1 consumed *vs.* the total food consumed (Food 1 + Food 2) minus 0.5 (corresponding to the non-preference threshold). The values were expressed between 0.5 and –0.5, where positive scores represented a preference for Food 1 (demonstrated food), negative scores for Food 2 and zero for no preference.

#### Social odor discrimination test

Social discrimination test, adapted from previously described procedures (41, 61), was conducted in a specific behavioral room under standard light-dark cycle. Residents and juvenile intruders (25–40 days old) male and female mice, were tested with an equivalent sex ratio. To characterize social memory performance, a Resident mouse is first placed in a clean cage at least 1 h before being tested. Then a mouse never encountered before (Intruder 1) is introduced into the cage for 3 min and the total social investigation time (general social exploration: oronasal, anogenital, nose-to-nose sniffing, allogrooming, chasing) performed by the Resident mouse is measured. 1 h after its removal, the same Intruder 1 is reintroduced into the cage and the social investigation time performed by the Resident mouse is again quantified during 3 min. Then, in order to assess social discrimination task performed by both genotypes, another intruder (Intruder 2) is presented rapidly to the Resident mouse (5 min after the end of the second session with Intruder 1) and the 3 min of social exploration time is similarly quantified.

### Statistics

ImageJ software (National Institute of Health, 1.48v) was used to analyze, in a stereological counting approach, the PS6 and c-Fos related positive cell density and mean intensity signals. An average of a minimum of two slices was used for the establishment of the global values per animal and a minimum of five animals were used per condition. GraphPad Prism 8.2.0 was used for statistical analysis and the generation of dot-plots graphics. Values are expressed as mean ± standard error of the mean (SEM). For innate odorant avoidance/attraction tests and food preference assays, mean responses significantly greater (innate odorant attraction and food preference) or lower (innate odorant avoidance) than 0 (corresponding to no preference) were considered as relevant. Consequently, their significances were assessed by one-tailed *Z*-test according to their respective mean *z*-values (preference ratio/SEM) (4). Fisher tests were used for evaluation of normality and homoscedasticity. Comparisons between conditions and genotypes were performed with one-tailed, two-tailed Student’s *t*-test or Wilcoxon *w*-tests accordingly. For social discrimination tests, the comparisons between Intruder sessions for homogenic greater or lower responses were assessed. Their significances were computed accordingly with one-tailed paired Student’s *t*-test. Significance levels are indicated as follows: #*p* < 0.05 for *z* values and: *p < 0.05, ^**^p < 0.01, ^***^p < 0.001 for *p*-values.

## Data availability statement

The original contributions presented in this study are included in the article/Supplementary material; the raw data are listed in the Supplementary Table 1; further inquiries can be directed to the corresponding authors.

## Ethics statement

This animal study was reviewed and approved by EXPANIM committee of the Lemanique Animal Facility Network and the veterinary authority of the Canton de Vaud (SCAV).

## Author contributions

AV, JB, and MCB designed the project and wrote the manuscript. AV, AL, AA, NG, MNT, DW, and JB carried out experimental procedures. AV, AL, AA, JB, and MCB analyzed the data. All authors discussed the results and approved the submitted version.
