# Supplementary material for: Food preference acquired by social transmission is altered by the absence of the olfactory marker protein in mice
Source: Front Nutr. 2022 Nov 9;9:1026373. doi: 10.3389/fnut.2022.1026373 (PMC9682023; doi:10.3389/fnut.2022.1026373)
Supplement: Supplementary file 1 [file Data_Sheet_1.pdf]

Supplementary materials

Food preference acquired by social transmission is altered by the absence of the olfactory marker protein in mice.

Aurélie de Vallière, Ana Catarina Lopes, Andrea Addorisio, Noah Gilliand, Monique Nenniger Tosato, Dean Wood, Julien Brechbühl and Marie-Christine Broillet.

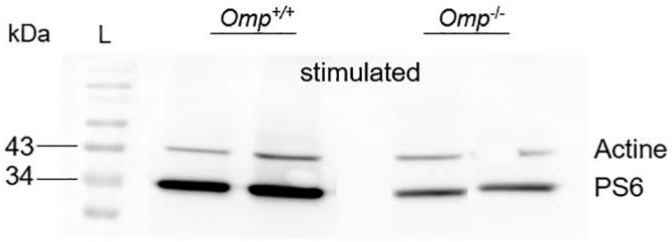

Supplementary Figure 1. Western blot analysis of phosphoserine 6 expression on MOE. PS6 expression (32 kDa) and actine expression (43 kDa). Intensity difference of PS6/actine in *Omp*$^{+/+}$ and *Omp*$^{-/-}$ mice stimulated here with Cocoa. L, ladder.

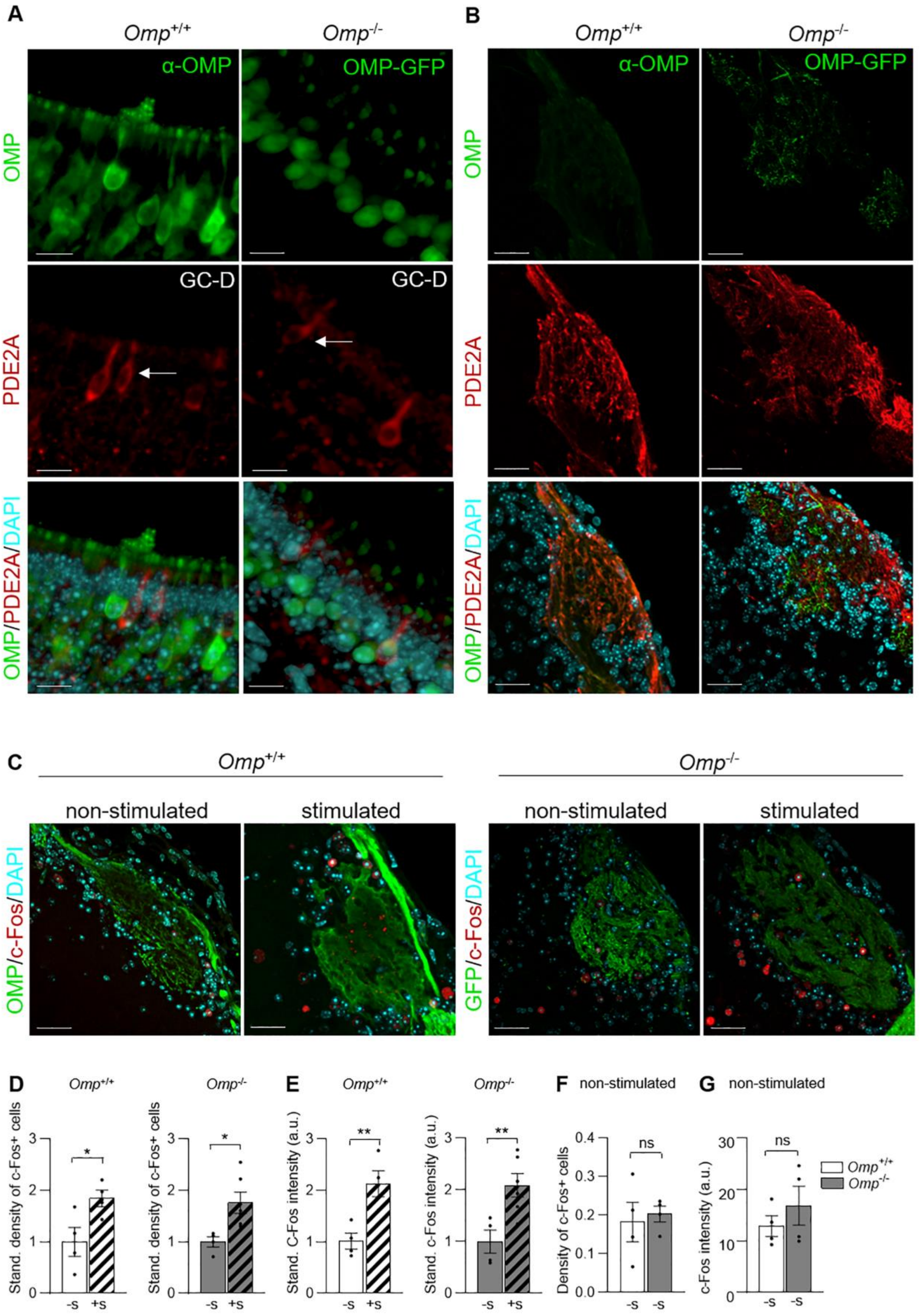
A
B
Omp+/+
Omp-/-
α-OMP
OMP-GFP
OMP
GC-D
PDE2A
OMP/PDE2A/DAPI
C
non-stimulated
stimulated
OMP/c-Fos/DAPI
GFP/c-Fos/DAPI
D
E
F non-stimulated
G non-stimulated
Stand. density of c-Fos+ cells
Stand. C-Fos intensity (a.u.)
Stand. c-Fos intensity (a.u.)
Density of c-Fos+ cells
c-Fos intensity (a.u.)
-s
+s
ns
*
**

Supplementary Figure 2. GC-D circuitry is present and functional in OMP null mice. GC-D circuitry is observed in both genotypes according to PDE2A stainings (red) in the MOE **(A)** and the OB **(B)**. (**C**) GC-D circuitry is functional in both genotypes according to c-Fos stainings (red) observed here in specific necklace glomeruli after CS_2 stimulation (+S). (**D-G**) Statistical quantification of c-Fos signal in the necklace glomeruli of both genotypes. In green (**A-C**), α-OMP showed the presence of OMP or its absence (GFP). In blue, the nuclei stained with DAPI. Scale bars: 10µm (**A**) and 30 µm (**B-C**). N = 3-6 animals per genotype. Values obtained are represented as mean ± SEM. Dot-plots representation in (**D-E**) are standardized to the non-stimulated condition (-S). For comparisons between genotypes, two-tailed Student's *t*-tests or Wilcoxon *w*-tests were used; for comparisons between condition (**D-E**), one-tailed paired Student's *t*-tests were used, *$p < 0.05$, **$p < 0.01$ and ns for non-significant.

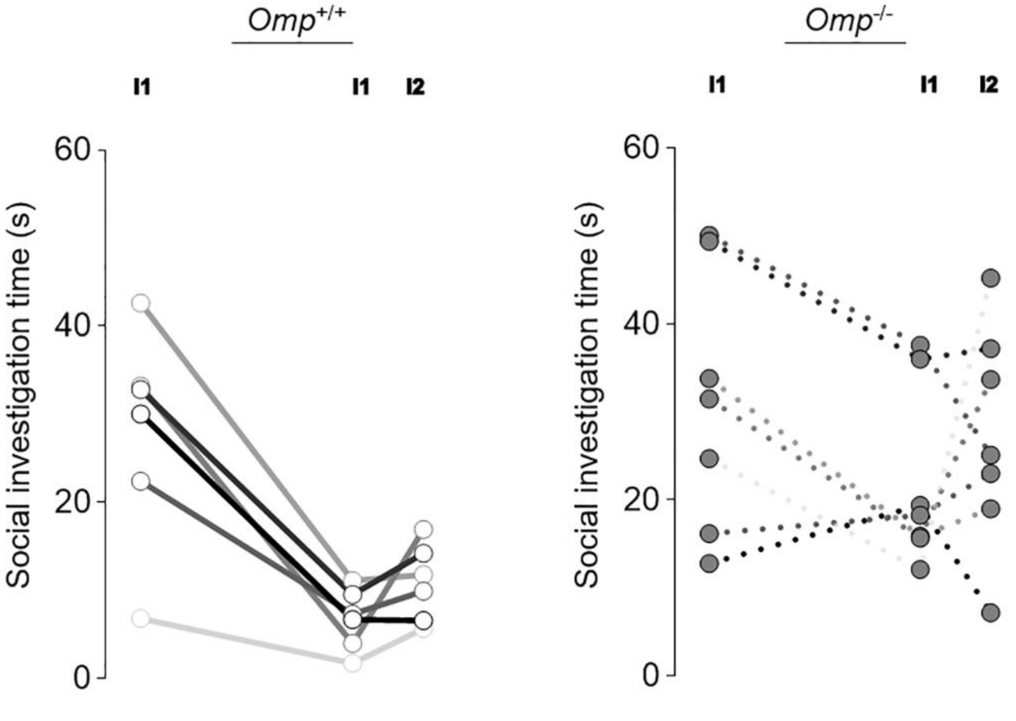

Supplementary Figure 3. Social memory and social discrimination in OMP null mice. Detailed of individual performances displayed by $Omp^{+/+}$ (continuous lines) and $Omp^{-/-}$ (dashed lines) mice during the social discrimination test (**Figure 4**). The unilateral trend displayed by mice between the two Intruder 1 trials (I1 – I1) indicates the development of a social memory. The unilateral trend observed in mice between the second Intruder 1 trial and the Intruder 2 trial (I1 – I2) reflects the ability for social discrimination. Thus, $Omp^{-/-}$ show alteration in social discrimination. N = 6-7 animals per genotype.
